# Recurrent malignant peripheral nerve sheath tumor presenting as an asymptomatic intravenous thrombus extending to the heart: a case report

**DOI:** 10.1186/s12957-021-02473-2

**Published:** 2022-01-07

**Authors:** Toru Hirozane, Robert Nakayama, Sayaka Yamaguchi, Tomoaki Mori, Naofumi Asano, Keisuke Asakura, Kazutaka Kikuta, Miho Kawaida, Aya Sasaki, Hajime Okita, Seishi Nakatsuka, Tsutomu Ito

**Affiliations:** 1 Department of Orthopedic Surgery, Keio University School of Medicine, 35 Shinanomachi, Shinjuku-ku, Tokyo, 160-8582 Japan; 2 Division of Thoracic Surgery, Department of Surgery, Keio University School of Medicine, Tokyo, Japan; 3 Division of Musculoskeletal Oncology and Orthopedic Surgery, Tochigi Cancer Center, Tochigi, Japan; 4 Department of Pathology, Keio University School of Medicine, Tokyo, Japan; 5 Department of Pathology, Tokyo Dental College Ichikawa General Hospital, Chiba, Japan; 6 Department of Radiology, Keio University School of Medicine, Tokyo, Japan; 7 Department of Cardiovascular Surgery, Keio University School of Medicine, Tokyo, Japan

**Keywords:** MPNST, Tumor thrombus, Venous invasion

## Abstract

**Background:**

Malignant peripheral nerve sheath tumor (MPNST) is a rare soft tissue sarcoma mainly treated via surgical resection. Herein, we report a case of MPNST wherein a massive tumor thrombus extended to the major veins and heart.

**Case presentation:**

A 39-year-old female with a history of neurofibromatosis type 1 developed MPNST from the right radial nerve. In addition to adjuvant chemotherapy, she underwent wide tumor resection and concomitant radial nerve resection, followed by postoperative radiotherapy. Histological evaluation revealed marked venous invasion. The 2-year follow-up CT revealed an asymptomatic recurrent tumor thrombus extending from the right subclavian vein to the heart. An urgent life-saving operation was performed to ligate the base of the right subclavian vein and remove the entire intravenous thrombus that extended to the right ventricle. The remaining tumor in the right subclavian vein increased in size 3 months after thrombectomy. After confirming the absence of any metastatic lesions, the patient underwent extended forequarter amputation to achieve surgical remission. One year later, a new metastasis to the right diaphragm was safely resected. The patient remains alive without any evidence of disease 2 years after the extended forequarter amputation.

**Conclusions:**

In cases of a previous history of microscopic venous invasion, recurrence can occur as a massive tumor thrombus that extends to the great vessels.

**Supplementary Information:**

The online version contains supplementary material available at 10.1186/s12957-021-02473-2.

## Background

Malignant peripheral nerve sheath tumors (MPNSTs) are rare, accounting for approximately 5% of all soft tissue sarcomas [1]. Previous studies have reported that 20–50% of patients with MPNST also have neurofibromatosis type 1 (NF1) [2, 3], which is an autosomal dominant condition with a birth incidence of approximately 1 in 2500 [4]. Patients with NF1 typically manifest café-au-lait macules in the first decade of life and cutaneous neurofibromas during adolescence [5], and they carry an increased risk of developing malignancies. MPNSTs occur in patients with NF1, with a cumulative lifetime risk of up to 10% [6]. Patients with NF1-MPNST reportedly have lower survival rates [7–9] and generally worse prognoses [7, 10] than those with sporadic MPNST. In the absence of metastases, the 5-year overall survival rates are 63% for sporadic MPNST and 33% for NF1-MPNST [10]. Furthermore, MPNSTs are traditionally chemotherapy insensitive. Radiotherapy provides local control and may delay recurrence onset but has little effect on long-term survival [1]. Since the clinical benefits of adjuvant therapy such as radiation or chemotherapy are limited in patients with MPNST [11], surgical resection is the usual treatment strategy.

Here, we present a unique case of a 39-year-old female with a previous history of MPNST from NF1, who developed an intravenous tumor thrombus that extended to the right ventricle. Urgent surgery for the intracardiac tumor thrombus, followed by subsequent second-stage extended forequarter amputation with partial chest wall resection, was successfully performed.

## Case presentation

This study was approved by the ethics committee of the Keio University School of Medicine. The patient provided signed informed consent for an Institutional Review Board-approved protocol for research use of medical records, pathologic specimens obtained as part of routine clinical care, and publication.

**Fig. 1:**
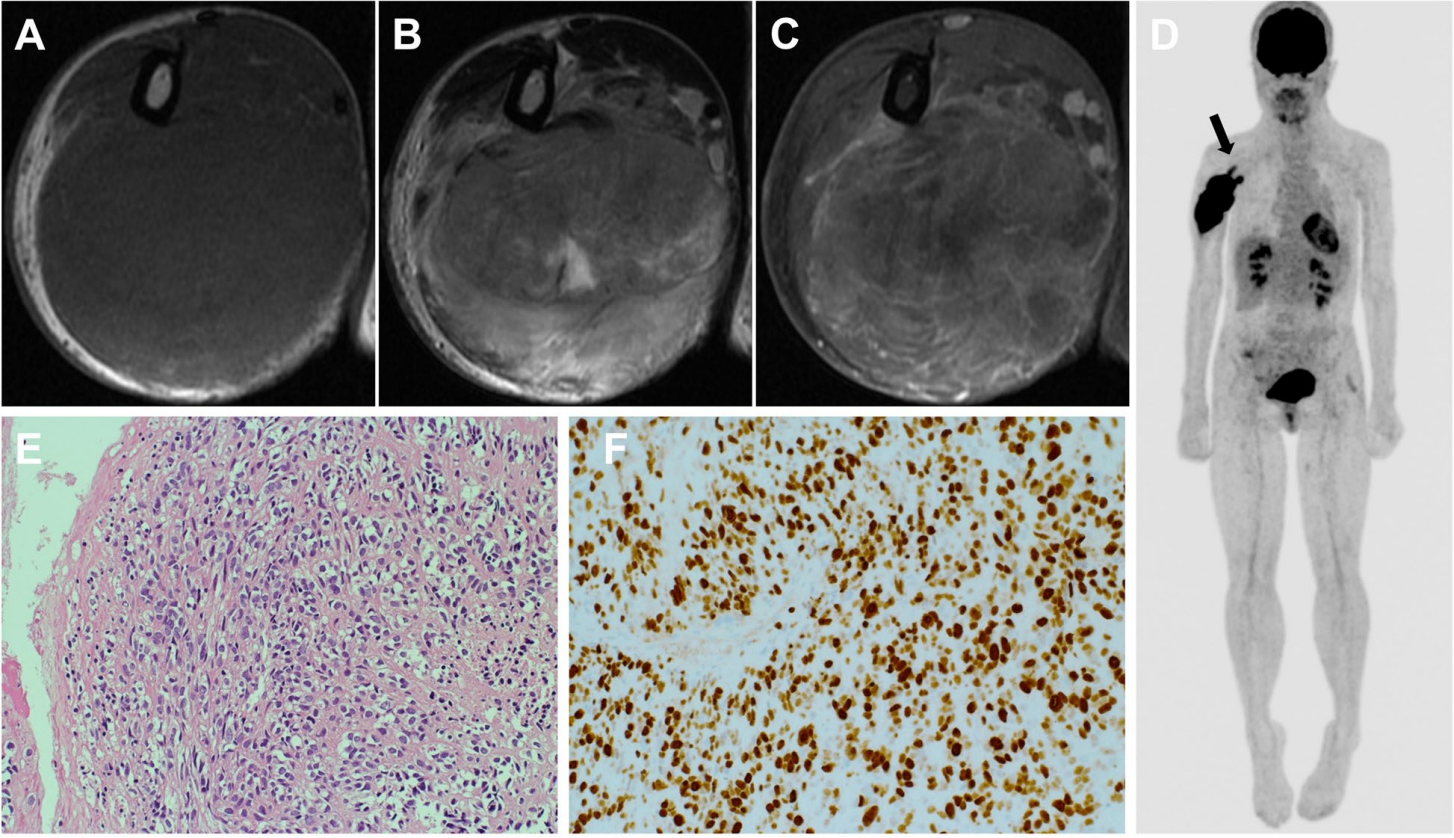
Imaging findings at presentation. **A**-**C** Axial MRI findings of the right upper extremity. The lesion had homogeneous low intensity on T1-weighted images (**A**), heterogeneous high intensity on T2-weighted images (**B**), and heterogeneous enhancement after gadolinium administration (**C**). **D** PET/CT demonstrates FDG uptake (SUVmax: 11.3) in a spindle-shaped tumor in her right arm. Two tail-like accumulations (black arrows) were found on the central side. **E**, **F** Pathological findings. A dense array of spindle-shaped tumor cells proliferated without forming an obvious structure (hematoxylin and eosin (**E**), ×40; staining with MIB-1 (**F**), ×40)

**Fig. 2:**
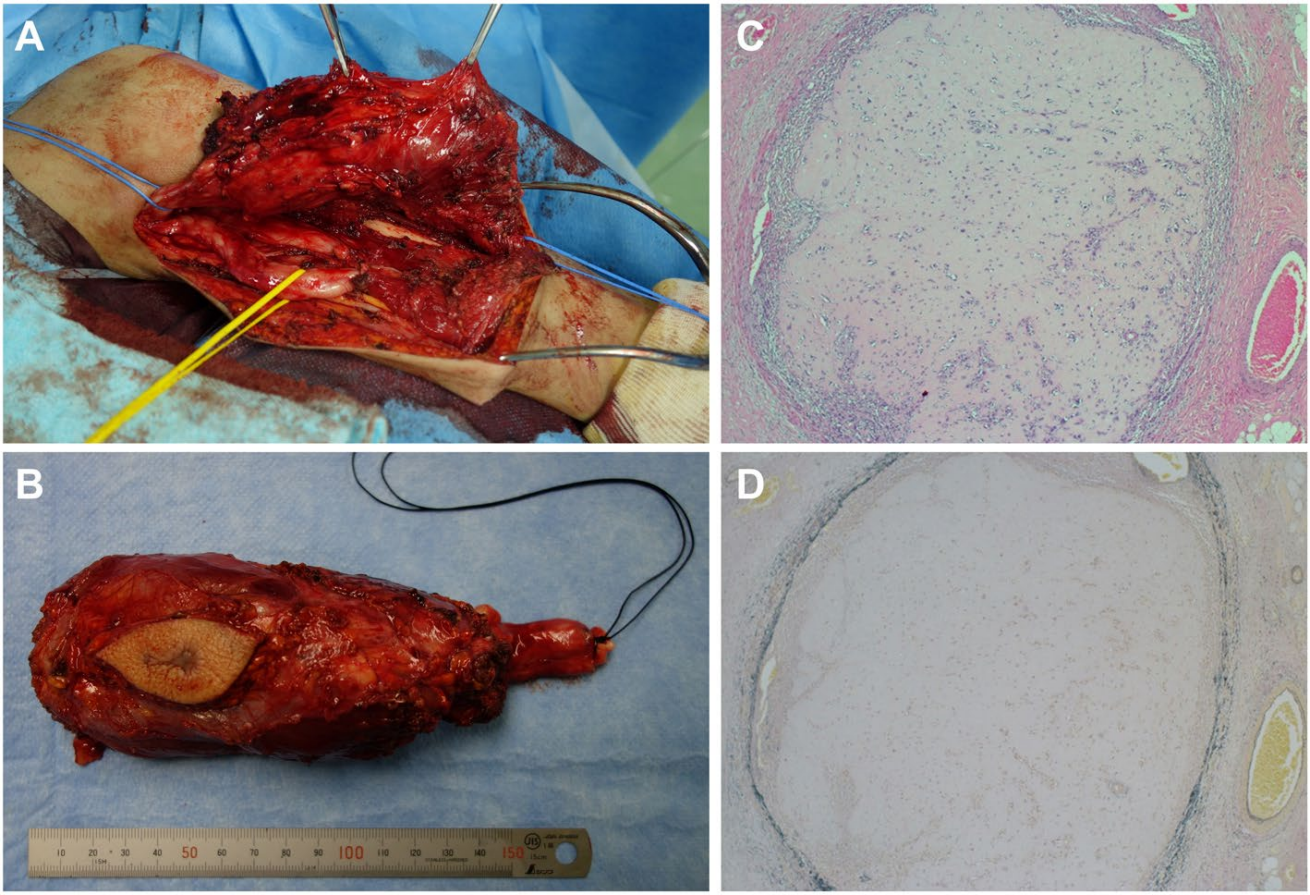
**A**, **B** Intraoperative findings. Wide resection of the tumor was performed. The tumor involved the radial nerve, whereas the ulnar nerve was preserved (**A**). The tumor was resected with radial nerve sacrifice (**B**). **C**, **D** Pathological findings. The tumor microscopically infiltrated the vessels (hematoxylin and eosin (**C**), ×40; staining with Elastica van Gieson (**D**), ×40)

The patient was a 39-year-old female with a history of NF1 who presented with an enlarging mass in the right arm with worsening radial nerve palsy. Her medical history included cervical kyphosis due to NF1. Magnetic resonance imaging (MRI) revealed a spindle-shaped tumor measuring 15 cm that almost completely occupied the posterior compartment of the upper arm (Fig. 1A-C). The tumor had homogeneous hypointensity on T1-weighted images, heterogeneous hyperintensity on T2-weighted images, and marked gadolinium enhancement on MRI (Fig. 1A-C). The patient underwent positron emission tomography (PET)/CT, which demonstrated high activity in the tumor and no metastatic disease. Two tail-like accumulations were observed on the proximal side, suggesting tumor extension along the nerve bundle (Fig. 1D). A biopsy confirmed high-grade MPNST with a MIB-1 index of 70% (Fig. 1E and F). Collectively, the patient was diagnosed with stage IIIA (T2N0M0, AJCC 8th edition) high-grade MPNST arising from the right radial nerve. She then received neoadjuvant chemotherapy (three courses of 60 mg/m^2^ doxorubicin plus 10 g/m^2^ ifosfamide) with excellent partial response and underwent wide resection with radial nerve sacrifice (Fig. 2A and B). The pathological examination revealed a negative surgical margin, a MIB-1 index of 10%, and marked microscopic vascular invasion (Fig. 2C and D). Thick neurofibromas were observed in both proximal and distal ends, and 90% of tumor necrosis had probably been due to the effects of the anti-cancer drugs. She then received postoperative radiotherapy and adjuvant chemotherapy (two courses of 60 mg/m^2^ doxorubicin plus 10 g/m^2^ ifosfamide).

**Fig. 3:**
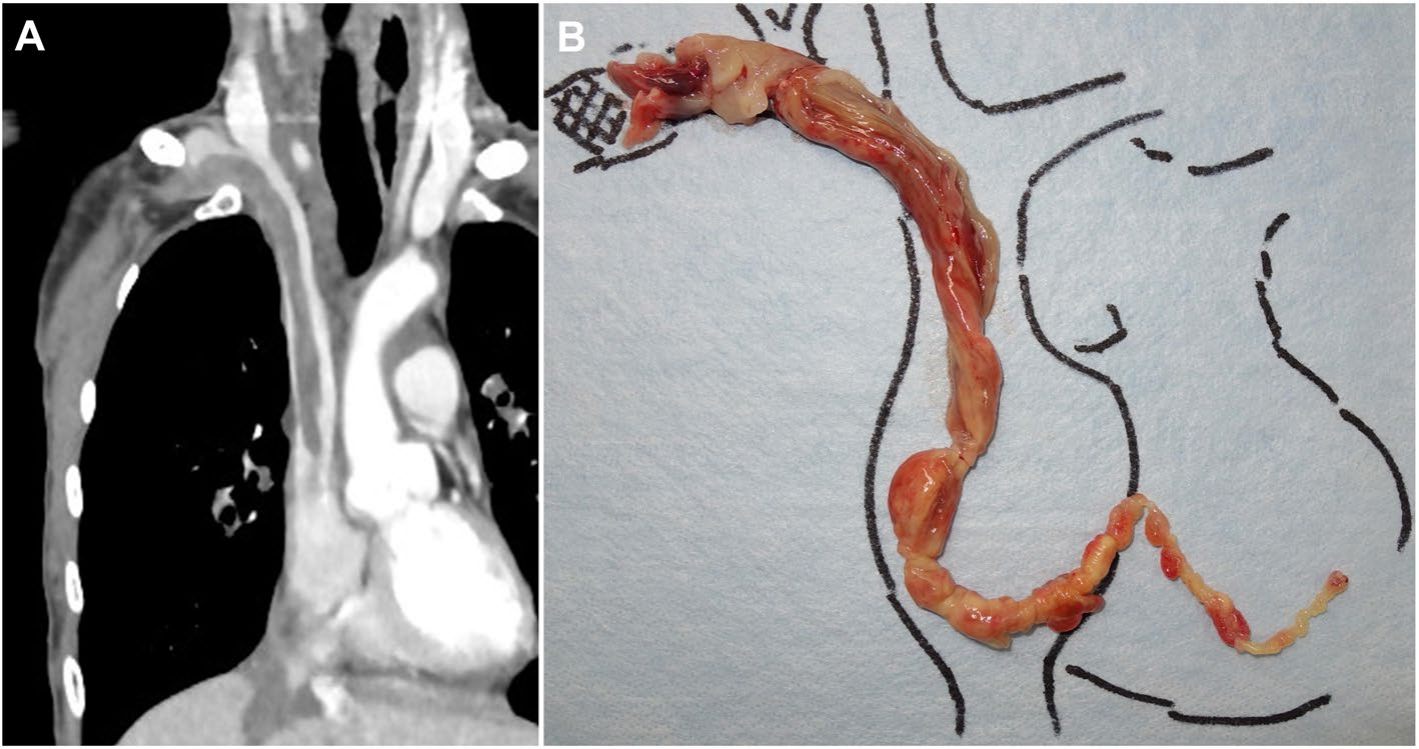
**A**, **B** Findings during the 2-year follow-up. A large thrombus extending to the right ventricle through the subclavian vein was detected on CT scan (**A**); surgical specimen of the intracardiac tumor thrombus (**B**)

**Fig. 4:**
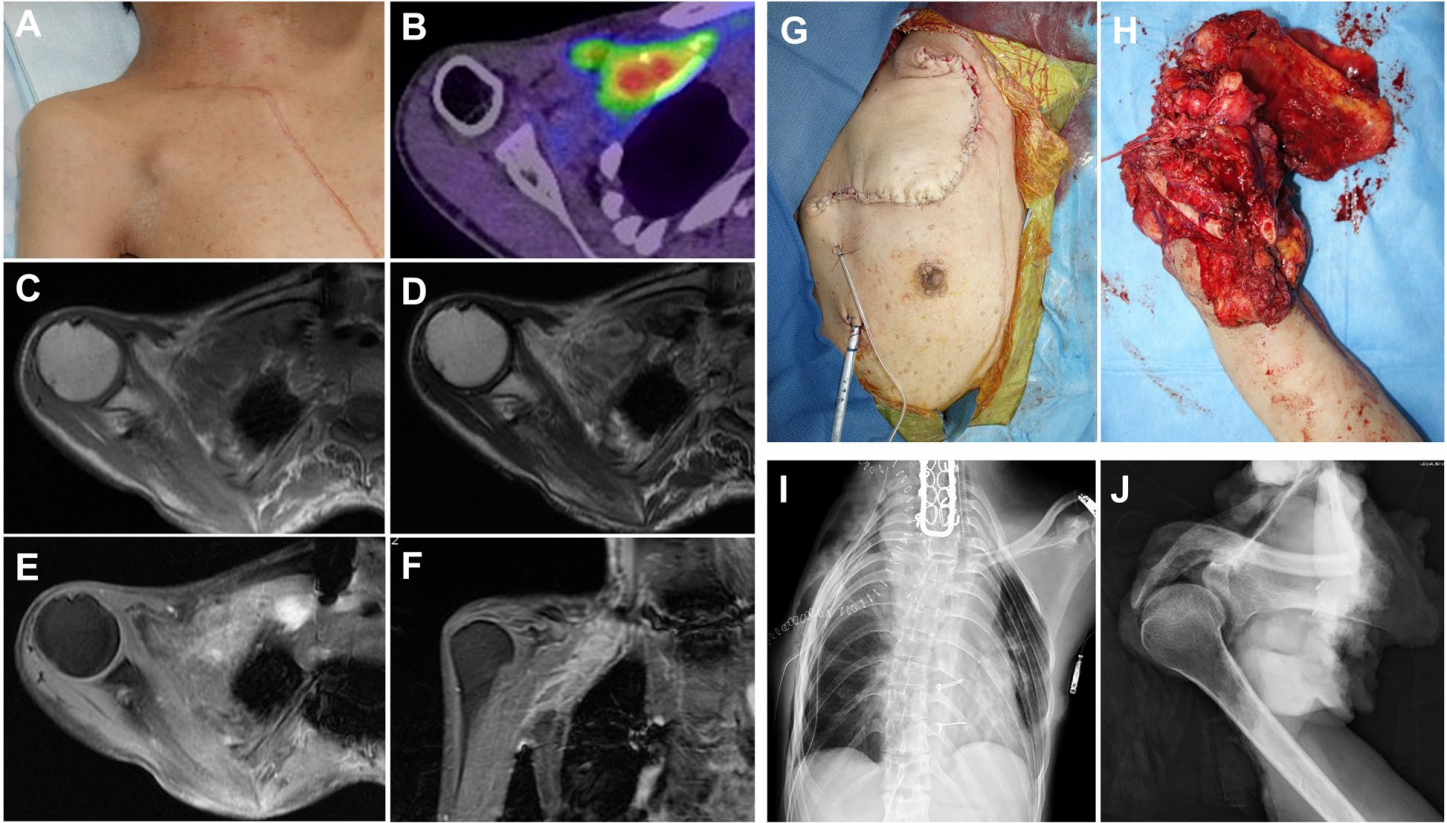
**A**-**F** Imaging findings 3 months after thrombectomy. Physical examination shows swelling in the right subclavicular lesion (**A**), and PET/CT of the chest demonstrates FDG uptake (SUVmax: 7.3) in the tumor (**B**). MRI shows a significant increase in the size of the remaining recurrent tumor in the subclavicular vein (T1-weighted axial (**C**), T2-weighted axial (**D**), fat-suppressed contrast-enhanced T1-weighted axial (**E**), and coronal (**F**) sections). **G**-**J** Postoperative pictures (**G** and **H**) and radiographs (**I** and **J**) of the performed forequarter amputation combined with partial chest wall resection

Two years after the initial surgery, a follow-up CT scan revealed an asymptomatic thrombus extending from the right subclavian vein to the right ventricle (Fig. 3A). Echocardiography verified the presence of a thrombus in the right atrium and right ventricle (Videos 1, 2), and a needle biopsy of the thrombus revealed MPNST recurrence. A diagnosis of localized MPNST recurrence was made because there was no obvious distant metastasis. Given the life-threatening nature of the tumor thrombus extending into a great vessel, urgent resection of the thrombus was performed. Under extracorporeal circulation, the tumor thrombus extending to the heart was removed from the right subclavian vein in a stepwise manner through three incisions in the right atrium, superior vena cava, and right subclavian vein, followed by ligation of the subclavian vein (Fig. 3B). However, the remaining distal part of the right subclavian vein was filled with a recurrent tumor. The patient was then carefully monitored through imaging studies to determine whether the local residual tumor would increase in size or if any metastatic lesions would appear. Three months after the thrombectomy, the remaining tumor in the right subclavian vein gradually increased in size (Fig. 4A) without any metastatic lesions. Because the enlarging recurrent MPNST was adjacent to the brachial plexus and the subclavian artery (Fig. 4B-F), we performed forequarter amputation combined with partial chest wall resection to achieve adequate surgical margins (Fig. 4G-J). Pathologically, the resected specimen exhibited histological findings similar to the needle biopsy specimen, with the proliferation of short spindle-shaped atypical cells with densely stained and enlarged nuclei, accompanied by hemorrhage and necrosis. Negative surgical margins were confirmed by pathological examination. Although the patient underwent surgery again for a newly found metastatic lesion on the right diaphragm 1 year later, the patient has been disease-free for 1 year since the last surgery.

## Discussion and conclusions

The clinical course of our case was highly unique in that the tumor recurred as an asymptomatic thrombus in the subclavian vein and had invaded the heart. Microscopic venous invasion during the initial surgery most likely led to an intravenous recurrence. Microscopic venous invasion is associated with a higher risk of recurrence and worse prognosis in cases such as pediatric adrenal cortical tumors, hepatocellular carcinomas, pancreatic cancers, and pancreatic neuroendocrine tumors [12–15]. A clinicopathological study of 24 patients with MPNST demonstrated vascular invasion as a poor prognostic factor [16]. However, few reports are available on recurrent MPNSTs with intravascular invasion. To the best of our knowledge, the present study is the first to report details on the presentation, diagnosis, and treatment of a case of MPNST with microscopic venous invasion extending to the heart. If histology suggests venous invasion, as it did in our case, there is a potential risk of intravenous recurrence and distant metastasis that can progress to a massive tumor thrombus extending to the heart, regardless of the tumor subtype.

Progression of malignant tumors to large vessels has been reported in renal and liver cancers, with a frequency of 3–20% [17–20]. Sarcomas with vascular tissue origins, such as intimal sarcoma [21] and angiosarcoma [22, 23], reportedly extend into large vessels. However, there are a few reports on sarcoma from non-vascular tissues extending into large vessels, including cases of low-grade endometrial stromal sarcoma [24], clear cell sarcoma of the kidney [25], osteosarcoma [26], and renal Ewing’s sarcoma [27]. MPNSTs extending to the great vessels are extremely rare, with only one other case reported in the literature [28]. This previous case presented a diagnostic challenge, and the final diagnosis was made only during the post-mortem examination.

Patients with cancer often have coagulation abnormalities and hence develop thromboses [29]. Although tumor thrombus is rare [30, 31], it is important to differentiate between a bland and a tumor thrombus among patients with cancer. Imaging evaluations such as CT, MRI, fluorodeoxyglucose on PET, and angiography [31] are useful; however, a biopsy is needed to definitively diagnose tumor thrombus in certain cases. Anticoagulant therapy such as heparin is usually ineffective, and surgical resection, chemotherapy, or radiation are, therefore, more common for tumor thromboses [30, 31]. In cases of renal cell carcinoma, complete surgical resection of a tumor thrombus has been reported to result in a 5-year survival rate > 50%, whereas incomplete tumor resection results in a 10% survival rate [31]. Technically, in areas where the proximal part of the involved vessels can be clamped or ligated, tumor thrombus removal includes procedures that prevent tumor cells from disseminating to the proximal side. In cases wherein a tumor thrombus involves the heart and where proximal blood flow cannot be disconnected, cardiopulmonary bypass is necessary. In our case, successfully removing the intracardiac tumor thrombus eventually enabled us to proceed with subsequent radical forequarter amputation for surgical remission.

MPNSTs derived from NF1 often occur in the major nerves, and radical wide resection of the affected nerves results in severe neurological deficits that lead to considerable physical impairment. If large MPNSTs occur in nerves adjacent to major arteries, such as the brachial plexus or femoral nerve, they require amputation at a higher level for radical resection. The frequency of amputation is reported to be 32% in deep and high-grade MPNSTs [32]. In patients with sarcoma, the frequency is reported to be 5% for primary disease and approximately 10% for recurrence [33]. Amputation is more frequent in MPNSTs than in other sarcomas because MPNSTs often occur in the major nerves, and amputation is recommended as a curative surgery to prevent recurrence [32]. If the tumor arises from the brachial plexus, forequarter amputation can be indicated [33–35]. Forequarter amputation involves removal of the arm and shoulder, including the scapula and part of the clavicle. If the tumor involves the chest wall, an extended procedure is performed, including resectioning a part of the chest wall, sometimes in combination with mastectomy or pneumonectomy. Seventy percent of the cases indicated for forequarter amputation are sarcomas, such as undifferentiated pleomorphic sarcoma, osteosarcoma, and synovial sarcoma, while the others are breast cancers and melanomas [35]. According to recent reports, the overall 5-year survival rate after forequarter amputation for malignant tumors of the shoulder girdle is approximately 20–40% [34–39] (Table 1). Complications after forequarter amputation include massive blood loss, wound necrosis, dissection, pneumonia, and atelectasis [34, 35, 38]. In addition, forequarter amputation has a massive impact on the psychological and functional integrity of patients [38]. We aim for the best clinical outcome for patients with NF1 and high-grade MPNST, and accordingly managed, in this case, to perform a wide (R0) resection of the primary tumor with radial nerve sacrifice and intensive adjuvant chemoradiotherapy. Three months after urgent removal of the life-threatening intracardiac tumor thrombus, the residual lesions in the remaining subclavian space increased in size. Given the absence of metastatic disease at the time and the extremely high risk of re-recurrence, we discussed the advantages of radical extended forequarter amputation over the less invasive chemoradiotherapy together with the patient, and she opted for the former. Although the patient underwent another surgery for a solitary metastasis and needed to be closely monitored, she is currently disease-free 2 years after the extended forequarter amputation, which was successfully managed by a multidisciplinary approach.

**Table 1:** Review of past reports with at least 20 patients who have undergone forequarter amputation

Reference	Year	***N***	Histology	Survival
Fanous et al. [36]	1976	21	15 sarcoma, 6 carcinoma	5 year OS 25% (C)
Bahgia et al. [34]	1997	20	18 sarcoma, 2 carcinoma	5 year OS: 21%, 30% (C)
Rickelt et al. [35]	2009	40	28 sarcoma, 10 carcinoma, 2 ulcer	5 year OS: 38% for all, 41% for sarcoma
Puhaindran et al. [37]	2012	26	Sarcoma & carcinoma	1 year OS 42 %
Elsner et al. [38]	2016	30	26 sarcoma, 4 carcinoma	5 year OS 39% (C)
Tsuda et al. [39]	2020	40	Bone sarcomas	5 year OS 30%
				C: Curative procedure

## Supplementary Information

**Additional file 1: Video 1**. Apical 4 chamber ultrasound view of the heart. A mobile tumor was detected in the right atrium and right ventricle.

**Additional file 2: Video 2**. Right ventricle focused color Doppler ultrasound view of the heart. The inflow to the right ventricle was almost unimpaired, but there was a mild tricuspid regurgitation.

## Data Availability

All relevant data are provided in the manuscript.

## Abbreviations

MPNST: Malignant peripheral nerve sheath tumor
NF1: Neurofibromatosis type 1
FDG: Fluorodeoxyglucose
PET: Positron emission tomography

## References

[1] Gupta G, Mammis A, Maniker A (2008). Malignant peripheral nerve sheath tumors. Neurosurg Clin N Am.

[2] Sorensen SA, Mulvihill JJ, Nielsen A (1986). Long-term follow-up of von Recklinghausen neurofibromatosis. Survival and malignant neoplasms. N Engl J Med.

[3] D’Agostino AN, Soule EH, Miller RH (1963). Primary malignant neoplasms of nerves (malignant neurilemomas) in patients without manifestations of multiple neurofibromatosis (Von Recklinghausen’s disease). Cancer.

[4] Huson SM, Compston DA, Clark P, Harper PS (1989). A genetic study of von Recklinghausen neurofibromatosis in south east Wales. I. Prevalence, fitness, mutation rate, and effect of parental transmission on severity. J Med Genet.

[5] National Institutes of Health Consensus Development Conference Statement: neurofibromatosis. Bethesda, Md., USA, July 13-15, 1987. Neurofibromatosis. 1988;1:172–8. 3152465

[6] Evans DG, Baser ME, McGaughran J, Sharif S, Howard E, Moran A (2002). Malignant peripheral nerve sheath tumours in neurofibromatosis 1. J Med Genet.

[7] Kolberg M, Holand M, Agesen TH, Brekke HR, Liestol K, Hall KS, Mertens F, Picci P, Smeland S, Lothe RA (2013). Survival meta-analyses for >1800 malignant peripheral nerve sheath tumor patients with and without neurofibromatosis type 1. Neuro Oncol.

[8] Anghileri M, Miceli R, Fiore M, Mariani L, Ferrari A, Mussi C, Lozza L, Collini P, Olmi P, Casali PG (2006). Malignant peripheral nerve sheath tumors: prognostic factors and survival in a series of patients treated at a single institution. Cancer.

[9] Stucky CC, Johnson KN, Gray RJ, Pockaj BA, Ocal IT, Rose PS, Wasif N (2012). Malignant peripheral nerve sheath tumors (MPNST): the Mayo Clinic experience. Ann Surg Oncol.

[10] Porter DE, Prasad V, Foster L, Dall GF, Birch R, Grimer RJ (2009). Survival in malignant peripheral nerve sheath tumours: a comparison between sporadic and neurofibromatosis type 1-associated tumours. Sarcoma.

[11] Bradford D, Kim A (2015). Current treatment options for malignant peripheral nerve sheath tumors. Curr Treat Options Oncol.

[12] Picard C, Orbach D, Carton M, Brugieres L, Renaudin K, Aubert S, Berrebi D, Galmiche L, Dujardin F, Leblond P (2019). Revisiting the role of the pathological grading in pediatric adrenal cortical tumors: results from a national cohort study with pathological review. Mod Pathol.

[13] Fujita N, Aishima S, Iguchi T, Mano Y, Taketomi A, Shirabe K, Honda H, Tsuneyoshi M, Oda Y (2011). Histologic classification of microscopic portal venous invasion to predict prognosis in hepatocellular carcinoma. Hum Pathol.

[14] Nanno Y, Toyama H, Otani K, Asari S, Goto T, Terai S, Ajiki T, Zen Y, Fukumoto T, Ku Y (2016). Microscopic venous invasion in patients with pancreatic neuroendocrine tumor as a potential predictor of postoperative recurrence. Pancreatology.

[15] Yamada M, Sugiura T, Okamura Y, Ito T, Yamamoto Y, Ashida R, Sasaki K, Nagino M, Uesaka K (2018). Microscopic venous invasion in pancreatic cancer. Ann Surg Oncol.

[16] Kar M, Deo SV, Shukla NK, Malik A, DattaGupta S, Mohanti BK, Thulkar S (2006). Malignant peripheral nerve sheath tumors (MPNST)--clinicopathological study and treatment outcome of twenty-four cases. World J Surg Oncol.

[17] Tse HF, Lau CP, Lau YK, Lai CL (1996). Transesophageal echocardiography in the detection of inferior vena cava and cardiac metastasis in hepatocellular carcinoma. Clin Cardiol.

[18] Cox SG, Davidson A, Thomas J, Brooks A, Hewitson J, Numanoglu A, Millar AJW (2018). Surgical management and outcomes of 12 cases of Wilms tumour with intracardiac extension from a single centre. Pediatr Surg Int.

[19] Adams LC, Ralla B, Bender YY, Bressem K, Hamm B, Busch J, Fuller F, Makowski MR (2018). Renal cell carcinoma with venous extension: prediction of inferior vena cava wall invasion by MRI. Cancer Imaging.

[20] Meyers D, Nixon NA, Franko A, Ng D, Tam VC. Tumour thrombus of the inferior vena cava extending into the right atrium in the setting of colon cancer. BMJ Case Rep. 2017;2017:bcr2016218107. 10.1136/bcr-2016-218107. 10.1136/bcr-2016-218107 PMC5318589 28193644

[21] Neuville A, Collin F, Bruneval P, Parrens M, Thivolet F, Gomez-Brouchet A, Terrier P, de Montpreville VT, Le Gall F, Hostein I (2014). Intimal sarcoma is the most frequent primary cardiac sarcoma: clinicopathologic and molecular retrospective analysis of 100 primary cardiac sarcomas. Am J Surg Pathol.

[22] Fatima J, Duncan AA, Maleszewski JJ, Kalra M, Oderich GS, Gloviczki P, Suri RM, Bower TC (2013). Primary angiosarcoma of the aorta, great vessels, and the heart. J Vasc Surg.

[23] Kakimoto T, Sasaki M, Morinaga S, Nakayama R, Minematsu N (2018). Asymptomatic pulmonary artery intimal sarcoma with chest wall metastasis as an initial manifestation: an autopsy case. Case Rep Med.

[24] Kudaka W, Inafuku H, Iraha Y, Nakamoto T, Taira Y, Taira R, Kamiya H, Tsubakimoto M, Totsuka Y, Kuniyoshi Y (2016). Low-grade endometrial stromal sarcoma with intravenous and intracardiac extension: a multidisciplinary approach. Case Rep Obstet Gynecol.

[25] Toyoda Y, Yamashita C, Sugimoto T, Yoshida M, Okada M (1998). Clear cell sarcoma of kidney with tumor extension into the right atrium. J Cardiovasc Surg (Torino).

[26] Hines N, Lantos G, Hochzstein J, Gitig A, DeAnda A (2007). Osteosarcoma of the lumbosacral spine invading the central venous pathways, right-sided cardiac chambers, and pulmonary artery. Skeletal Radiol.

[27] Rizzo D, Barone G, Ruggiero A, Maurizi P, Furfaro IF, Castagneto M, Riccardi R (2011). Massive venous thrombosis of inferior vena cava as primary manifestation of renal Ewing’s sarcoma. Clin Nephrol.

[28] Chapman EM, Hanelin J (1948). Neurofibrosarcoma, with invasion of right innominate vein and superior vena cava. N Engl J Med.

[29] Dos Santos AA, Baumgratz JF, Vila JH, Castro RM, Bezerra RF (2016). Clinical and surgical strategies for avoiding or reducing allogeneic blood transfusions. Cardiol Res.

[30] Pao TH, Hsueh WT, Chang WL, Chiang NJ, Lin YJ, Liu YS, Lin FC (2019). Radiotherapy for inferior vena cava tumor thrombus in patients with hepatocellular carcinoma. BMC Cancer.

[31] Quencer KB, Friedman T, Sheth R, Oklu R (2017). Tumor thrombus: incidence, imaging, prognosis and treatment. Cardiovasc Diagn Ther.

[32] Vauthey JN, Woodruff JM, Brennan MF (1995). Extremity malignant peripheral nerve sheath tumors (neurogenic sarcomas): a 10-year experience. Ann Surg Oncol.

[33] Clark MA, Thomas JM (2003). Amputation for soft-tissue sarcoma. The Lancet Oncology.

[34] Bhagia SM, Elek EM, Grimer RJ, Carter SR, Tillman RM (1997). Forequarter amputation for high-grade malignant tumours of the shoulder girdle. J Bone Joint Surg Br.

[35] Rickelt J, Hoekstra H, van Coevorden F, de Vreeze R, Verhoef C, van Geel AN (2009). Forequarter amputation for malignancy. Br J Surg.

[36] Fanous N, Didolkar MS, Holyoke ED, Elias EG (1976). Evaluation of forequarter amputation in malignant diseases. Surg Gynecol Obstet.

[37] Puhaindran ME, Chou J, Forsberg JA, Athanasian EA (2012). Major upper-limb amputations for malignant tumors. J Hand Surg Am.

[38] Elsner U, Henrichs M, Gosheger G, Dieckmann R, Nottrott M, Hardes J, Streitburger A (2016). Forequarter amputation: a safe rescue procedure in a curative and palliative setting in high-grade malignoma of the shoulder girdle. World J Surg Oncol.

[39] Tsuda Y, Fujiwara T, Evans S, Kaneuchi Y, Abudu A (2020). Extra-articular resection of shoulder joint for bone sarcomas: oncologic and limb-salvage outcomes of 32 cases compared with shoulder disarticulation and forequarter amputation. J Surg Oncol.

